# Healthy People 2020: Social Determinants of Cigarette Smoking and Electronic Cigarette Smoking among Youth in the United States 2010–2018

**DOI:** 10.3390/ijerph17207503

**Published:** 2020-10-15

**Authors:** Gang Wang, Liyun Wu

**Affiliations:** 1 School of Journalism and Communication, Wuhan University, Wuhan 430072, China; wangucb@whu.edu.cn; 2 Ethelyn R. Strong School of Social Work, Norfolk State University, 700 Park Avenue, Norfolk, VA 23504, USA

**Keywords:** youth, cigarette smoking, electronic cigarette smoking, Healthy People 2020, social determinant of health, National Health Interview Survey (NHIS)

## Abstract

The purpose of this study was to determine social determinants of cigarette smoking and ever using electronic cigarettes (e-cigarettes) among young adults aged 18 to 25 years in the United States between 2010 and 2018. Using secondary data from National Health Interview Surveys (NHIS) across the 2010, 2014, and 2018 survey years, this study analyzed the prevalence rates of cigarette smoking and ever using e-cigarettes between 2010 and 2018, demographic and socioeconomic disparities in smoking, and the relationship between previous e-cigarette use and current smoking. First, the past decade witnessed a notable decline in conventional cigarette smoking and a sharp increase in e-cigarette use among youth. These trends were consistent regardless of socioeconomic status. Second, demographic and socioeconomic disparities persisted in cigarette smoking. Non-Hispanic white male youth were more likely to become smokers as they grew older. Young people with lower educational attainment, living below the U.S. federal poverty level, and having a poor physical health status had a higher smoking prevalence. Third, previous e-cigarette use was more likely to relate to subsequent cigarette use among young people. To achieve the Healthy People 2020 objectives, tobacco control programs and interventions need to be more specific in higher prevalence groups and service providers should not assume that there is a one-size-fits-all model for youth.

## 1. Introduction

By the end of 2017, the United States had over 34 million current cigarette smokers, 14% of U.S. adults, who smoked either every day or some days during the typical week, a significant decline from 20.9% in 2005 [1]. This prevalence rate was ten percent among young adults aged 18 to 24 years. Despite the falling prevalence of smoking, cigarette smoking still has significant public health consequences. In a report which summarized 50 years of progress in tobacco control and prevention, the Office of the Surgeon General [2] specified that more than 16 million Americans suffered from a disease caused by smoking. Many diseases, 87 percent of lung cancer deaths, 32 percent of coronary heart disease deaths, and 79 percent of all cases of chronic obstructive pulmonary disease, were caused by smoking, and smoking has become the leading cause of preventable disease and death (about 1 in 5 deaths) in the United States [3].

The large prevalence of tobacco use has become one of the twelve leading health indicators put forward by Healthy People 2020, which was led by the U.S. Surgeon General and provided a national agenda for improving the health of the nation and achieving health equity for the period 2010–2019 [4]. Healthy People was launched in 1980 by the U.S. Surgeon General to provide 10-year national objectives for improving health nationwide. Since its inception, the nation has witnessed four decades of Healthy People initiatives, including Healthy People 1990, Healthy People 2000, Healthy People 2010, and Healthy People 2020. The fifth iteration of Healthy People, Healthy People 2030, is launched in 2020 and sets national health objectives for the next ten years 2020–2030.

Given the fact that tobacco use has significant public health consequences in relation to a wide range of physical, psychiatric, and behavior health measures, it is imperative to understand the causes and determinants of cigarette smoking. According to the Office of Disease Prevention and Health Promotion (ODPHP) [5], social determinants of health refers to “conditions in the environment in which people are born, live, learn, work, play, worship, and age that affect a wide range of health, functioning, and quality-of-life outcomes and risks.” Research findings based on adult samples have provided empirical evidence about the social determinants of health factors (low socioeconomic status, unequal distribution of resources, services, and power) and subsequent inequalities in tobacco use [6].

The past decade has witnessed rapidly changing patterns of tobacco use among youth and young adults. According to the U.S. Surgeon General [7], e-cigarettes have emerged as the most frequently used tobacco product among youth in the United States in 2014, surpassing conventional cigarettes. E-cigarettes are new forms of tobacco products that contain nicotine and users consume it via an inhaled aerosol. The e-cigarette market has produced a rapidly emerging and diversified product class with several interchangeable names, “e-cigs”, “e-hookahs”, “mods”, “vape pens”, “vapes”, and “tank systems”. Research findings based on adult samples have indicated that delivery of nicotine doses from e-cigarettes can be as large as or larger than conventional cigarettes [8,9]. A group of experts of the National Academies of Sciences, Engineering, and Medicine (2018) released a report to summarize the public health consequences of e-cigarettes [10]. There were forty-seven major conclusions, including eight pieces of conclusive evidence, ten of substantial evidence, eight of moderate evidence, twelve of limited evidence, four of insufficient evidence, and five with no available evidence in terms of diseases and harm related to e-cigarettes. In addition, there are few studies on the health effects of long-term use of e-cigarettes or the association between use of e-cigarettes and cigarette smoking [11]. Given the relatively new introduction of e-cigarettes, new scientific evidence of their health effects continues to emerge. This study fills a gap by examining the associations between previous e-cigarette consumption and current cigarette smoking among youth and young people. The purpose of this study was three-fold: (a) to estimate the prevalence rates of cigarette and e-cigarette smoking among different demographic and socioeconomic groups among young adults aged 18 to 25 years, (b) to estimate socioeconomic disparities in smoking, and (c) to determine the relationship between previous e-cigarette use and current cigarette smoking.

## 2. Materials and Methods

### 2.1. Study Design, Data and Sample

This study utilized the secondary data from the National Health Interview Survey (NHIS) across three waves to study the trend and prevalence of cigarette smoking and electronic cigarette smoking as well as social determinants of smoking among young adults in the past decade. Since 1957, the U.S. Census Bureau has been the data collection agency for the NHIS and it uses household-based sampling to collect data on an annual basis among the non-institutionalized U.S. civilian population nationwide. The data mainly collected are information on health status, health care access, and progress which are frequently used as major data sources to evaluate national health objectives [12]. Because the year 2018 was the latest dataset available for public use, this study utilized data from these three waves, 2010, 2014, and 2018, to represent the health trend in the past decade. Out of these three survey waves, data in 2010 represent the beginning of the decade, data in 2014 represent the middle point of the decade, and data in 2018 represent the end point of the decade. The inclusion criterion had resulted in samples which only included young adults aged 18 to 25 years old. For each wave of data, the NHIS included several data files such as person file, sample adult file, and family file. Using the unique person identifier, the authors merged person, sample adult, and family files into one combined dataset for each wave. The sample sizes for three waves were 3281 respondents in 2010, 3981 in 2014, and 2195 in 2018, respectively.

The NHIS datasets are available for download from the Centers for Disease Control and Prevention (CDC) website for public use. All the downloadable public use data files are not individually identifiable, and there is no way to link data with the subjects from whom the data were originally collected. Therefore, this study did not involve human subjects and was not subject to approval from the Institutional Review Board (IRB) office.

### 2.2. Measurements

Smokers were identified using two major variables. Current cigarette smokers were those who reported having smoked every day or some days of the typical week at the time of survey. The indicator for current smoker was measured as 1 if the respondent smoked every day or some days, and 0 if not smoking at all. Ever electronic cigarette smokers were those who ever smoked electronic cigarettes at the time of survey. The indicator for an ever e-cigarette smoker was measured as 1 if the respondent ever used electronic cigarettes and 0 if never. Although current cigarette smokers were measured each wave, ever e-cigarette smokers were only measured in recent waves 2014 and 2018. Former smokers were excluded from analyses of smoking status because there were overlaps between current smoking and former smoking status.

Several sociodemographic variables were measured in the study of disparities in smoking prevalence. Demographic variables included age, gender, and race/ethnicity. Age was continuous in actual years old. Gender was dichotomous with 1 = male and 2 = female. Race/ethnicity was coded as four groups: 1 = Non-Hispanic white, 2 = Non-Hispanic black, 3 = Hispanic, and 4 = other. Socioeconomic variables included education, income-to-poverty ratio, self-reported health status, and citizenship status. Education and income are considered as two most important indicators of socioeconomic status. Based on the original variable, “R’s highest schooling”, education was recoded into four categories: 1 = less than high school, 2 = high school or General Education Development (GED) equivalent, 3 = some college or associate degrees, 4 = bachelor and above. The income-to-poverty ratio was created to proxy the family’s financial security and the poverty threshold took into account both income and family size. In general, a ratio less than one implied that the income was less than the set poverty threshold and those people whose incomes fell under the threshold were considered poor and in poverty. The income-to-poverty ratio was measured in three categories: 1 = less than 100% U.S poverty level, 2 = between 100% and 199%, and 3 = 200% and above. The self-reported health status was a categorical variable, where the categories had an intrinsic order with 1 = excellent, 2 = very good, 3 = good, and 4 = fair/or poor. The citizenship indicated whether respondents had U.S. citizenship with 1 = yes and 0 = no.

### 2.3. Analyses

Descriptive statistics were reported for all variables across three survey waves. In addition to frequencies and proportions, the line charts were drawn to represent the trends over time. Logistic regressions were conducted to test the relationship between sociodemographic factors, being a previous electronic cigarette smoker, and being a current smoker. Odds ratios and 95% confidence intervals were reported. All the statistical analyses were completed by using SPSS version 24.0, IBM North America, New York, United States.

## 3. Results

### 3.1. Descriptive Statistics of Demographics

A total of 9457 respondents were included in this study: 3281 respondents in 2010, 3981 respondents in 2014, and 2195 respondents in 2018. Table 1 presents the sample characteristics of respondents by year. Young adults were between 18 to 25 years old and as age increased, more observations were included. There were more females than males in 2010 and the gender distribution was even in the 2014 and 2018 waves. Non-Hispanic White accounted for half or more than half of the sample (49.3% in 2010, 56.3% in 2014, and 57.0% in 2018). Non-Hispanic Black represented one sixth or one seventh of the sample (17.6% in 2010, 14.4% in 2014, and 12.5% in 2018). Hispanic youth were oversampled, making up about one quarter of the sample (25.7% in 2010, 21.1% in 2014, and 20.6% in 2018), and other race/ethnicity represented less than ten percent (7.4%, 8.2%, 9.9%). As for educational attainment, the majority of the sample had some college or associate degrees (40.8% in 2010, 44.0% in 2014, and 40.9% in 2018). Less than one fifth had four-year college degrees or above (15.2% in 2010, 16.7% in 2014, and 21.6% in 2018), and forty percent had high school education or below (44.1% in 2010, 39.3% in 2014, and 37.5% in 2018). There was a significant portion of the sample who were poor or in poverty: 34.9% less than 100% income-to-poverty ratio in 2010, 34.2% in 2014, and 22.9% in 2018. The majority of the sample participants self reported their health status as excellent, very good, or good. There was only a small percentage of them in poor or fair health conditions (5.0% in 2010, 4.7% in 2014, and 4.1% in 2018). Ninety percent of the sample were U.S. citizens (88.1% in 2010, 90.6% in 2014, and 91.6% in 2018).

### 3.2. Prevalence of Current Cigarette Smoking and Electronic Cigarette Smoking

**Figure 1:**
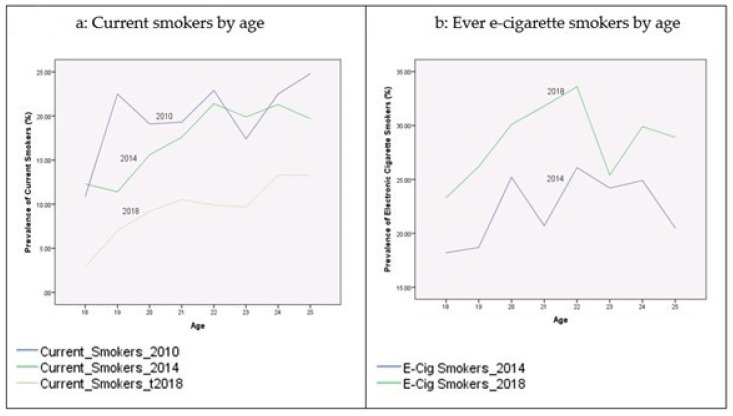
Prevalence of smoking behaviors by age, NHIS 2010–2018.

**Figure 2:**
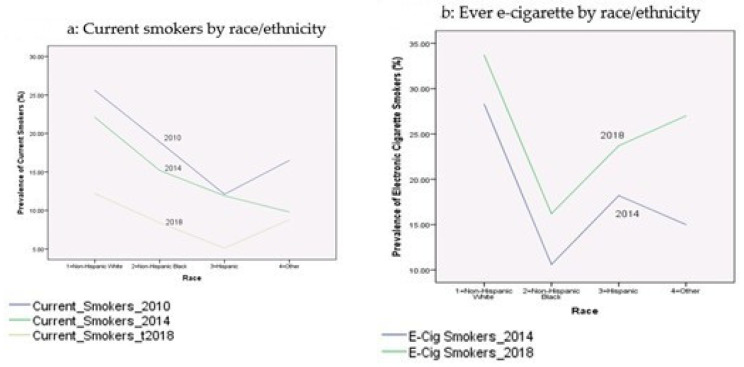
Prevalence of smoking behaviors by race/ethnicity, NHIS 2010–2018.

**Figure 3:**
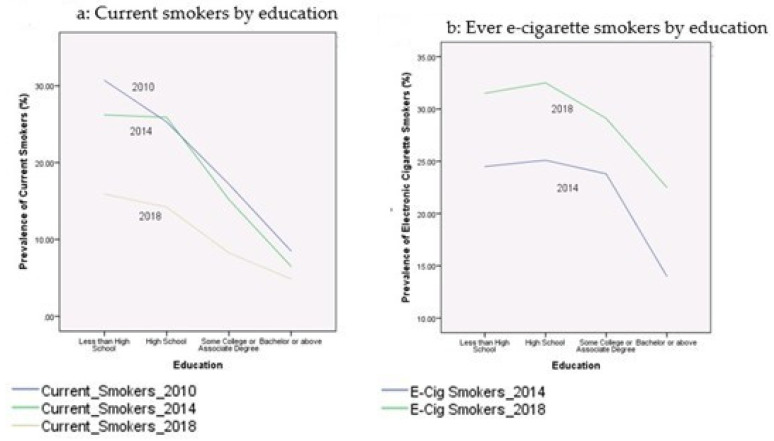
Prevalence of smoking behaviors by education, NHIS 2010–2018.

**Figure 4:**
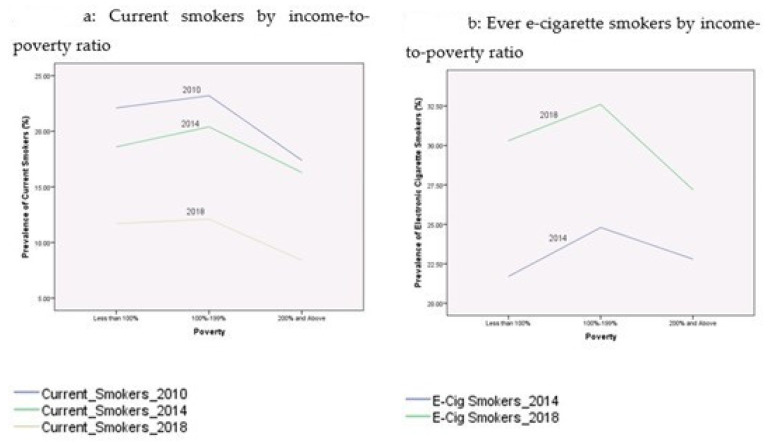
Prevalence of smoking behaviors by income-to-poverty ratio, NHIS 2010–2018.

**Figure 5:**
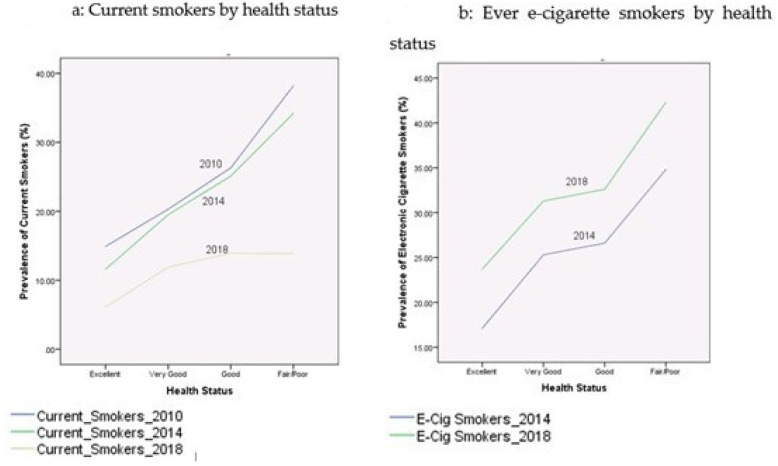
Prevalence of smoking behaviors by health status, NHIS 2010–2018.

Table 2 presents the prevalence rates of current cigarette smokers and electronic cigarette smokers among youth during the period 2010–2018. These proportions were broken down by age, gender, race/ethnicity, education, income-to-poverty ratio, reported health status, and citizenship status. Figure 1, Figure 2, Figure 3, Figure 4 and Figure 5 depict these trends in terms of line graphs. The trends were consistent across different sociodemographic indicators. Specifically speaking, the prevalence rates of cigarette smoking declined in the past decade with the prevalence rate highest in 2010 and lowest in 2018. In a sharp contrast, electronic cigarette smokers increased from 2014 to 2018.

### 3.3. Predicting the Likelihood of Smoking Behaviors

Table 3 shows the results from logistic regression predicting the likelihood of being a current cigarette smoker by controlling sociodemographic variables and previous electronic cigarette smoking status. Each column reports respective results from each wave. As mentioned above, electronic cigarette data were not collected in 2010, therefore the ever e-cigarette indicators were only included in the analyses for two survey years, 2014 and 2018. In both waves, after controlling sociodemographic factors, previous electronic cigarette smokers in 2014 (*p* < 0.001, OR = 10.428, 95% CI 8.502–12.790) and 2018 (*p* < 0.001, OR = 6.666, 95% CI 4.770–9.316) were more likely to be current smokers, compared to non-smokers. Demographic characteristics also strongly predicted the likelihood of being a current cigarette smoker versus a non-smoker. As youth grew older, they were more likely to become smokers and the results were statistically significant across three waves in 2010 (*p* < 0.001, OR = 1.193, 95% CI 1.141–1.248), 2014 (*p* < 0.001, OR = 1.191, 95% CI 1.136–1.248), and 2018 (*p* < 0.001, OR = 1.285, 95% CI 1.188–1.389). There were more male youth smoking cigarettes than female youth in 2010. No gender difference in smoking behaviors was identified in years 2014 and 2018. There were significant racial/ethnic differences among cigarette smokers. Compared to other race/ethnicity, there were more non-Hispanic white youth smokers in 2014 (*p* < 0.01, OR = 1.869, 95% CI 1.198–2.916). There were fewer non-Hispanic black youth smokers in 2010 (*p* = 0.024, OR = 0.590, 95% CI 0.373–0.932), and fewer Hispanic youth smokers in both 2010 (*p* < 0.001, OR = 0.315, 95% CI 0.199–0.498) and 2018 (*p* < 0.05, OR = 0.495 95% CI 0.245–1.001).

In addition, the prevalence of smoking in youth varied widely in terms of their socioeconomic status. Compared to youth who had bachelor or graduate degrees, youth without four-year college education were significantly more likely to smoke cigarettes and the lower the education, the higher likelihood of smoking. In regard to income-to-poverty ratio, lower income was significantly associated with higher likelihood of smoking in 2010 and 2018. Youth whose families were living below the 100% poverty line had the highest likelihood of being current smokers (*p* = 0.008, OR = 1.363, 95% CI 1.083–1.716) in 2010 and (*p* = 0.016, OR = 1.621, 95% CI 1.095–2.400) in 2018. Youth whose families lived between the 100% and 200% poverty lines also had a higher likelihood of being current smokers (*p* = 0.005, OR = 1.428, 95% CI 1.115–1.829) in 2010, compared to youth whose family incomes were above 200% income-to-poverty ratio. The likelihood of smoking also varied widely according to youth’s self-reported physical health status. Compared to those who reported excellent health, youth whose health statuses were very good/good/fair/poor were more likely to become current smokers and the fair/poor sample had the highest likelihood of smoking (*p* < 0.001, OR = 2.883, 95% CI 1.943–4.277) in 2010. Youth who were U.S. citizens were significantly more likely to smoke cigarettes than immigrant youth in 2010 (*p* < 0.001, OR = 2.135, 95% CI 1.425–3.199).

## 4. Discussion

This study examined a population-based national sample of U.S. young adults whose ages were between 18 and 25 years old, in three survey years from 2010 to 2018. Research findings reported that prevalence rates of smoking behaviors varied widely across sociodemographic characteristics and there were strong and significant relationships between previous electronic cigarette smoking and current smoking status. The proportion of smokers in the US had significantly decreased during the period 2010 to 2018, and this trend was consistent regardless of age, gender, race/ethnicity, education, income-to-poverty ratio, self-reported health status, and citizenship. This declining trend among U.S. youth is similar to findings by the CDC [13], stating that only ten percent of young adults aged 18 to 24 years smoked cigarettes in 2017. This falling trend is also similar to that of other countries such as Argentina and Brazil, to name a few [14,15]. Youth were more likely to become cigarette smokers as they grew older. There were more male smokers than female smokers. Non-Hispanic white youth were significantly more likely to be smokers compared to other racial/ethnic youth. Socioeconomic status played an important role in predicting the likelihood of being a smoker. Citizens were associated with a higher prevalence of cigarette smoking compared to immigrant youth. Youth who had lower levels of educational attainment, who lived below or near the U.S. federal poverty levels, and who had poor physical health status were very likely to become current smokers. These findings were consistent with previous research on cigarette smoking among people of low socioeconomic status [16,17]. Studies by Carlson et al. (2018) identified barriers to quitting smoking among youth with a low socioeconomic status and concern about weight gain was statistically associated with quitting status. These findings also suggested that risks associated with cigarette smoking may be evident as these social determinants clearly resulted in negative health outcomes.

There are important implications based on the findings of this study. First, comprehensive tobacco control policies, anti-smoking campaigns and educational programs may have contributed to the notable decline in cigarette smoking among young people in the past decade. Since 1965, the U.S. Surgeon General has been generating annual reports on smoking and health which inform the general public of the health consequences of smoking and changes in the tobacco landscape. More than half of the fifty U.S. states and a growing number of cities and counties have enacted strong smoke-free laws that require workplaces and public places such as restaurants and bars to be smoke-free [18]. These tight government regulations successfully created barriers for smokers and reduced the prevalence of smoking. In addition, most commercial health insurance plans and Medicaid insurance were required to provide coverage on smoking cessation programs to help smokers to quit smoking [19]. In addition to these nationwide laws and initiatives, numerous anti-smoking campaigns and educational and prevention programs gave young people tools and resources to prevent smoking at early ages. Second, while the notable decline in smoking rates is a public health success, the socioeconomic inequality in cigarette smoking prevalence rates is still astonishing. In order to narrow the health disparity gap, tobacco control initiatives need to be more specific by targeting groups with higher smoking prevalence. For youth whose families are of low socioeconomic status, social service and health care providers might provide culturally competent smoking cessation programs for different racial/ethnic youth because they have different cultures and environments as regards cigarette smoking. Providing culturally competent programs may become an effective approach in smoking cessation and prevention. As smoking is a learned and socially-mediated behavior [20], youths are heavily influenced by social norms in their immediate circle and are very likely to experiment with tobacco use if family members or friends smoke. Third, in sharp contrast to the decline in conventional cigarette smoking, use of electronic smoking is dramatically rising among youth. Although unsafe health effects of e-cigarette use among U.S. youth and young adults are clearly documented by the CDC [21], there are no studies on the long-term effects of e-cigarette use due to the fact that e-cigarettes are new nicotine-delivery products on the market. The investigation of lung injury associated with use of e-cigarettes will continue. Fourth, since Healthy People 1990 was first made public by the U.S. Surgeon General in 1979, Healthy People initiatives have been consistently providing overarching health objectives and targets for four decades (Healthy People 1990, 2000, 2010, and 2020). While each decade was unique, achieving health equity and eliminating health disparities were consistent and coherent health objectives across four decades. As indicated by findings from this study, cigarette prevalence rates are still unevenly distributed and widely varied across demographic and socioeconomic status. Out of the five key areas of social determinants of health, this study analyzed three areas, namely, education, economic stability, health and health care, and did not touch the other two areas, social and community context, neighborhood and built environment. Therefore, future research has to continue exploring other key areas of social determinants of smoking and identifying effective approaches to prevent tobacco use.

There are several limitations of this study that deserve additional attention. First, this study used a repeated cross-sectional design by examining changes in trends over time in the past decade. Unlike a longitudinal cohort study which represents changes within the same individuals, this cross-sectional study is a snapshot and prevents examination of the longitudinal association between social determinants and current smoking behaviors. Thus, this study cannot establish a causal relationship between social determinants and smoking prevalence. Second, this study utilized data from three survey years—2010, 2014, and 2018, to represent the past decade. Once the 2019 wave data are available, researchers will be able to extend this study by using data from 2010–2019 to represent the time trend during the past decade. With that said, 2010 represents the beginning of this decade, 2015 is the middle point of the decade, and 2019 is the end of the decade. Third, data were limited to youth aged 18–25 and therefore cannot be generalized to other age groups.

Despite methodological limitations, this study significantly contributed to bridging the gap in knowledge about trends in smoking over the last decade, demographic and socioeconomic disparities in smoking, and associations between previous electronic cigarette usage and current smoking status among U.S. young people aged 18 to 25 years. Using comprehensive and representative national-level survey data, the results of this study can shed light on the social determinants of smoking prevalence among young people worldwide.

## 5. Conclusions

This was the first study to examine the social determinants of cigarette smoking prevalence and associations between previous electronic cigarette usage and current cigarette smoking among youth aged 18 to 25 in nationally-representative U.S. samples during the period 2010 to 2018. Although overall smoking prevalence has decreased in the U.S. over the past decade (2010–2018), socio-economic disparities related to cigarette smoking still persist. Young people with lower educational attainment, living below the U.S. federal poverty level, and having poor physical health status had a higher smoking prevalence. Youth who previously smoked electronic cigarettes were more likely to become current smokers. To achieve the Healthy People objectives of achieving health equity and eliminating health disparities, tobacco control programs and interventions need to be more specific and service providers should not assume that there is a one-size-fits-all model for youth with dramatically different demographic and socioeconomic characteristics. Interventions need to be sensitive and specific in higher prevalence groups such as youth with lower social economic status. There is still room for improvement. As young adulthood represents a distinct developmental period of the life course, early intervention and prevention programs for smoking cessation will significantly reduce adverse effects and benefit society as a whole. Moreover, public health policies, regulations, and prevention programs need to prioritize strategies which address the shifting trends toward electronic cigarette usage among young adults.

## Figures and Tables

**Table 1:** Sociodemographic Statistics, National Health Interview Survey 2010–2018.

Variables	2010	2010	2014	2014	2018	2018
	Frequency	Proportion	Frequency	Proportion	Frequency	Proportion
Age						
18	311	9.50%	373	9.40%	210	9.60%
19	355	10.80%	396	9.90%	215	9.80%
20	361	11.00%	437	11.00%	250	11.40%
21	383	11.70%	482	12.10%	256	11.70%
22	442	13.50%	509	12.80%	292	13.30%
23	482	14.70%	574	14.40%	319	14.50%
24	467	14.20%	582	14.60%	315	14.40%
25	480	14.60%	628	15.80%	338	15.40%
Gender						
Male	1532	46.70%	1955	49.10%	1103	50.30%
Female	1749	53.30%	2026	50.90%	1092	49.70%
Race/Ethnicity						
Non-Hispanic White	1619	49.30%	2241	56.30%	1251	57.00%
Non-Hispanic Black	576	17.60%	573	14.40%	274	12.50%
Hispanic	844	25.70%	840	21.10%	453	20.60%
Other race group	242	7.40%	327	8.20%	217	9.90%
Education						
Less than high school	535	16.40%	478	12.00%	239	10.90%
High school/GED	905	27.70%	1085	27.30%	584	26.60%
Some college	1335	40.80%	1751	44.00%	896	40.90%
Bachelor and above	497	15.20%	664	16.70%	474	21.60%
Income-to-Poverty ratio						
Less than 100%	1057	34.90%	1309	34.20%	488	22.90%
100–199%	725	23.90%	923	24.10%	480	22.60%
200% and greater	1249	41.20%	1599	41.70%	1160	54.50%
Reported health status						
Excellent	1319	40.20%	1676	42.10%	935	42.60%
Very good	1098	33.50%	1330	33.40%	723	32.90%
Good	696	21.20%	788	19.80%	436	19.90%
Fair/Poor	165	5.00%	187	4.70%	90	4.10%
Citizenship status						
Yes	2883	88.10%	3603	90.60%	2011	91.60%
No	390	11.90%	376	9.40%	184	8.40%
Observations	3281	3281	3981	3981	2195	2195

**Table 2:** The Prevalence Rates of Current Cigarette Smokers vs. Electronic Cigarettes Smokers–National Health Interview Surveys (NHIS) 2010–2018.

Variables	Current Smokers 2010 (95% CI)	Current Smokers 2014 (95% CI)	Current Smokers 2018 (95% CI)	Ever Electronic Smokers 2014 (95% CI)	Ever Electronic Smokers 2018 (95% CI)
Age					
18	10.9%	12.3%	2.90%	18.20%	23.30%
	(7% to 14%)	(9% to 16%)	(1% to 5%)	(14% to 22%)	(18% to 29%)
19	22.5%	11.4%	7.00%	18.70%	26.20%
	(18% to 27%)	(8% to 15%)	(4% to 10%)	(15% to 26%)	(20% to 32%)
20	19.1%	15.6%	9.20%	25.20%	30.10%
	(15% to 23%)	(12% to 19%)	(6% to 13%)	(21% to 29%)	(24% to 36%)
21	19.3%	17.6%	10.50%	20.70%	31.80%
	(15% to 23%)	(14% to 21%)	(7% to 14%)	(17% to 24%)	(26% to 38%)
22	22.9%	21.4%	9.90%	26.10%	33.60%
	(19% to 27%)	(18% to 25%)	(6% to 13%)	(22% to 30%)	(28% to 39%)
23	17.4%	19.9%	9.70%	24.20%	25.40%
	(14% to 21%)	(17% to 23%)	(6% to 13%)	(21% to 28%)	(21% to 30%)
24	22.5%	21.3%	13.30%	24.90%	29.90%
	(19% to 26%)	(18% to 25%)	(10% to 17%)	(21% to 28%)	(35% to 28%)
25	24.8%	19.7%	13.30%	20.50%	28.90%
	(21% to 29%)	(17% to 23%)	(10% to 17%)	(17% to 24%)	(24% to 34%)
Gender					
Male	22.9%	19.9%	11.60%	27.20%	35.50%
	(21% to 25%)	(18% to 22%)	(10% to 14%)	(25% to 29%)	(33% to 38%)
Female	18.0%	16.1%	8.20%	18.10%	22.10%
	(16% to 20%)	(14% to 18%)	(7% to 10%)	(16% to 20%)	(20% to 25%)
Race/Ethnicity					
Non-Hispanic White	25.6%	22.1%	12.20%	28.30%	33.70%
	(24% to 28%)	(20% to 24%)	(10% to 14%)	(26% to 30%)	(31% to 36%)
Non-Hispanic Black	18.9%	15.2%	8.40%	10.60%	16.20%
	(16% to 22%)	(12% to 18%)	(5% to 12%)	(8% to 13%)	(12% to 21%)
Hispanic	12.1%	11.9%	5.10%	18.20%	23.70%
	(10% to 14%)	(10% to 14%)	(3% to 7%)	(16% to 21%)	(20% to 28%)
Other race group	16.5%	9.8%	8.80%	15.00%	27.00%
	(12% to 21%)	(7% to 13%)	(5% to 13%)	(11% to 19%)	(21% to 33%)
Education					
Less than high school	30.7%	26.2%	15.90%	24.50%	31.50%
	(27% to 35%)	(22% to 30%)	(11% to 21%)	(21% to 28%)	(26% to 37%)
High school/GED	25.3%	25.9%	14.20%	25.10%	32.50%
	(22% to 28%)	(23% to 29%)	(11% to 17%)	(22% to 28%)	(29% to 36%)
Some college	17.2%	15.2%	8.30%	23.80%	29.10%
	(15% to 19%)	(14% to 17%)	(6% to 10%)	(22% to 26%)	(26% to 32%)
Bachelor and above	8.5%	6.5%	4.90%	14.00%	22.50%
	(6% to 11%)	(5% to 8%)	(3% to 7%)	(11% to 17%)	(19% to 26%)
Income-to-Poverty ratio					
Less than 100%	22.1%	18.6%	11.70%	21.70%	30.30%
	(20% to 25%)	(17% to 21%)	(9% to 15%)	(19% to 24%)	(26.2% to 34.4%)
100%–199%	23.2%	20.4%	12.10%	24.80%	32.60%
	(20% to 26%)	(18% to 23%)	(9% to 15%)	(22% to 28%)	(28% to 37%)
200% and greater	17.4%	16.3%	8.40%	22.80%	27.20%
	(15% to 19%)	(14% to 18%)	(7% to 10%)	(21% to 25%)	(25% to 30%)
Reported health status					
Excellent	14.9%	11.6%	6.10%	17.10%	23.70%
	(13% to 17%)	(10% to 13%)	(5% to 8%)	(15% to 19%)	(21% to 26%)
Very good	20.3%	19.5%	11.90%	25.30%	31.30%
	(18% to 23%)	(17% to 22%)	(10% to 14%)	(23% to 28%)	(28% to 35%)
Good	26.3%	25.1%	13.90%	26.60%	32.60%
	(23% to 30%)	(22% to 28%)	(11% to 17%)	(24% to 30%)	(28% to 37%)
Fair/Poor	38.2%	34.2%	13.90%	34.80%	42.30%
	(31% to 46%)	(27% to 41%)	(7% to 21%)	(28% to 42%)	(32% to 52%)
Citizenship status					
Yes	21.6%	18.7%	10.10%	23.80%	29.70%
	(20% to 23%)	(17% to 20%)	(9% to 11%)	(22% to 25%)	(28% to 32%)
No	10.8%	10.9%	8.20%	10.90%	19.00%
	(8% to 14%)	(8% to 14%)	(4% to 12%)	(8% to 14%)	(13% to 25%)
Observations (n)	3281	3981	2195	3981	2195

**Table 3:** Predicting Smoking Behaviors by Using Logistic Regressions, NHIS 2010–2018.

	Being a Current Smoker	Being a Current Smoker	Being a Current Smoker
	2010	2014	2018
	Col (1)	Col (2)	Col (3)
	Odds Ratio	Odds Ratio	Odds Ratio
	(Lower to Upper 95% CI)	(Lower to Upper 95% CI)	(Lower to Upper 95% CI)
Ever electronic cigarette smoker		10.428 (8.502 to 12.790) ***	6.666 (4.770 to 9.316) ***
Age	1.193 (1.141 to 1.248) ***	1.191 (1.136 to 1.248) ***	1.285 (1.188 to 1.389) ***
Gender			
Male	1.408 (1.162 to 1.706) ***	1.028 (0.841 to 1.255)	1.141 (0.828 to 1.571)
Female (reference)			
Race/Ethnicity			
Non-Hispanic White	1.177 (0.780 to 1.778)	1.869 (1.198 to 2.916) **	1.475 (0.824 to 2.643)
Non-Hispanic Black	0.590 (0.373 to 0.932) *	1.502 (0.906 to 2.492)	1.130 (0.543 to 2.352)
Hispanic	0.315 (0.199 to 0.498) ***	0.702 (0.433 to 1.140)	0.495 (0.245 to 1.001) *
Other race group (ref)			
Education			
Less than high school	10.227 (6.644 to 15.742) ***	7.540 (4.828 to 11.776) ***	6.729 (3.560 to 12.718) ***
High school/GED	6.056 (4.102 to 8.942) ***	6.782 (4.585 to 10.031) ***	4.416 (2.591 to 7.526) ***
Some college	3.242 (2.223 to 4.728) ***		2.339 (1.386 to 3.947) ***
Bachelor and above (ref)		2.698 (1.853 to 3.929) ***	
Income-to-Poverty ratio			
Less than 100%	1.363 (1.083 to 1.716) **	1.173 (0.927 to 1.484)	1.621 (1.095 to 2.400) *
100%–199%	1.428 (1.115 to 1.829) **	1.147 (0.892 to 1.475)	1.466 (0.999 to 2.152)
200% and greater (ref)			
Reported health status			
Excellent (ref)			
Very good	1.578 (1.252 to 1.988) ***	1.654 (1.303 to 2.098) ***	1.759 (1.202 to 2.575) *
Good	1.888 (1.461 to 2.439) ***	2.094 (1.606 to 2.730) ***	1.618 (1.054 to 2.482) *
Fair/Poor	2.883 (1.943 to 4.277) ***	2.420 (1.606 to 3.648) ***	1.419 (0.714 to 2.818)
Citizenship status			
Yes	2.135 (1.425 to 3.199) ***	1.040 (0.683 to 1.582)	1.236 (0.912 to 1.674)
No (ref)			
Observations (n)	3281	2195	3981
−2 Log likelihood	2695.38	2650.297	

* *p* < 0.05; ** *p* < 0.01; *** *p* < 0.001.
